# Research on the quality evaluation of crude drugs

**DOI:** 10.1007/s11418-024-01845-8

**Published:** 2024-10-03

**Authors:** Hiroyuki Fuchino

**Affiliations:** 1 Research Center for Medicinal Plant Resources, Tsukuba Division, National Institutes of Biomedical Innovation, Health and Nutrition, 1-2 Hachimandai, Tsukuba City, Ibaraki, 305-0843 Japan; 2 Department of Pharmacy, Niigata University of Pharmacy and Medical and Life Sciences, 265-1 Higashijima, Akiha-Ku, Niigata City, Niigata, 956-8603 Japan

**Keywords:** Crude drug, Processing, Liquid chromatography-nuclear magnetic resonance/mass spectrometry

## Abstract

**Abstract:**

As crude drugs are natural products, their quality may vary. However, the degradation of the active ingredients in the compositional changes that occur during processing and preparation also affects the medicinal properties of the Kampo formula, which uses herbal medicines; therefore, a detailed investigation of the effects of compositional changes during preparation is required. Plant constituents vary in content depending on the year of cultivation and the plant part; however, detailed studies have rarely been reported for some crude drugs. Liquid chromatography-nuclear magnetic resonance/mass spectrometry revealed the degradation process of saponins, which are unstable components of the crude drug “Achyranthes root.” The presence of diterpenes unstable with respect to drying temperature in the leaves of the crude drug “Leonurus herb” was revealed and their structures were elucidated. At the examination stage of the degradation process of perillaldehyde, the characteristic aromatic component of Perilla herb, it was elucidated that some specimens contained a small amount of perillaldehyde and that they contained more α-asarone. A trend toward lower ephedrine content was observed toward the tip of the above-ground branching of the Ephedra herb. Multivariate analysis was also introduced into the quality assessment of crude drugs and was established as a tool to identify bioactive compounds using the component diversity of crude drugs and to elucidate component differences due to the cultivation environment.

**Graphic abstract:**

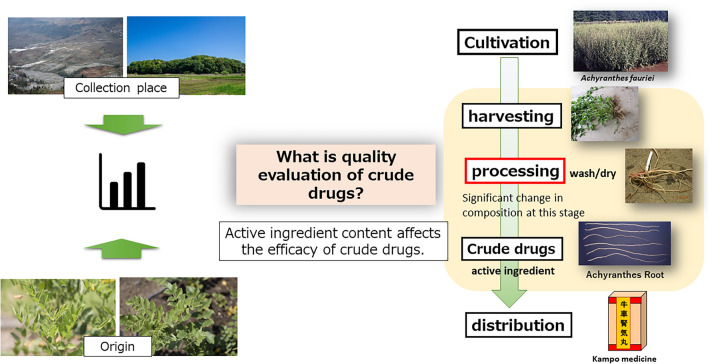

## Introduction

As crude drugs are natural products, their quality may vary. In addition, the compositional changes that occur during processing and preparation after harvesting the original plant, such as degradation of the active ingredients, affect the medicinal properties of Kampo formulae, which uses crude drugs; therefore, a detailed investigation of the effects of compositional changes during processing and preparation is required. Crude drugs are traditionally processed using a variety of methods, depending on the crude drug. A typical example of traditional processing is “processed aconite root,” the original plant of which, *Aconitum carmichaeli*, contains aconitine alkaloids, which are highly poisonous, but the knowledge of our ancestors has enabled it to be used as a crude drug by reducing its toxicity by steaming and preserving it in salt [1]. Traditionally, the roots of Japanese angelica are bundled and dried in the sun, followed by squeezing in hot water before drying [2]. This processing increases market value by making the roots more flexible by adding more starch and removing soil sediment and worm eggs from complicated roots. However, the changes in the composition of these crude drugs during processing and preparation have not been studied in detail. For example, drying is an essential process in the production of crude drugs, and despite the importance of the relationship between drying temperature and compositional changes, no detailed studies on this have been conducted. Depending on the drying temperature, enzymatic reactions may proceed, and enzymatic hydrolysis of glycosides may occur, whereas higher temperatures may lead to chemical pyrolysis as well as the loss of volatile components. Considering these factors, a balanced set of conditions is necessary to produce high-quality crude drugs. In addition, the phytochemical constituent content varies depending on the number of years the original based plant has been cultivated. Although some crude drugs have been empirically recognized as suitable for a certain number of years of cultivation, there are no clear data regarding this. The uneven distribution of constituents also needs to be considered as inconsistent quality can lead to confusion in medical practice. This review outlines research on the quality assessment of crude drugs.

## Decomposition processes of the unstable components of crude drugs

### Investigation of the unstable components of Achyranthes root using liquid chromatography-nuclear magnetic resonance/mass spectrometry (LC-NMR/MS) [3]

The crude drug, Achyranthes root (牛膝), originates from the roots of *Achyranthes fauriei*, and although there are many reports on the constituents of Achyranthes root, in addition to the long-known ecdysteroids such as inokosterone [4], specific dicarboxylic acids bound to the sugar moiety, such as Triterpene saponins, have been reported. The latter was first isolated from *A. fauriei* by Ida et al. in the form of methyl esters and named achyranthoside [5], whereas Yoshikawa et al. isolated the same compound from *Beta vulgaris* in the form of a free carboxylic acid and named it betavulgaroside [6]. These saponins have a structure in which a specific dicarboxylic acid is attached via an acetal to the sugar moiety attached to the hydroxyl group at C-3 of the oleanane skeleton and are considered to be relatively unstable. Thus, many unstable saponins and achyranthosides are present immediately after being harvested from the field; however, structural analysis of unstable components requires separation, purification, and immediate spectral analysis.

**Fig. 1:**
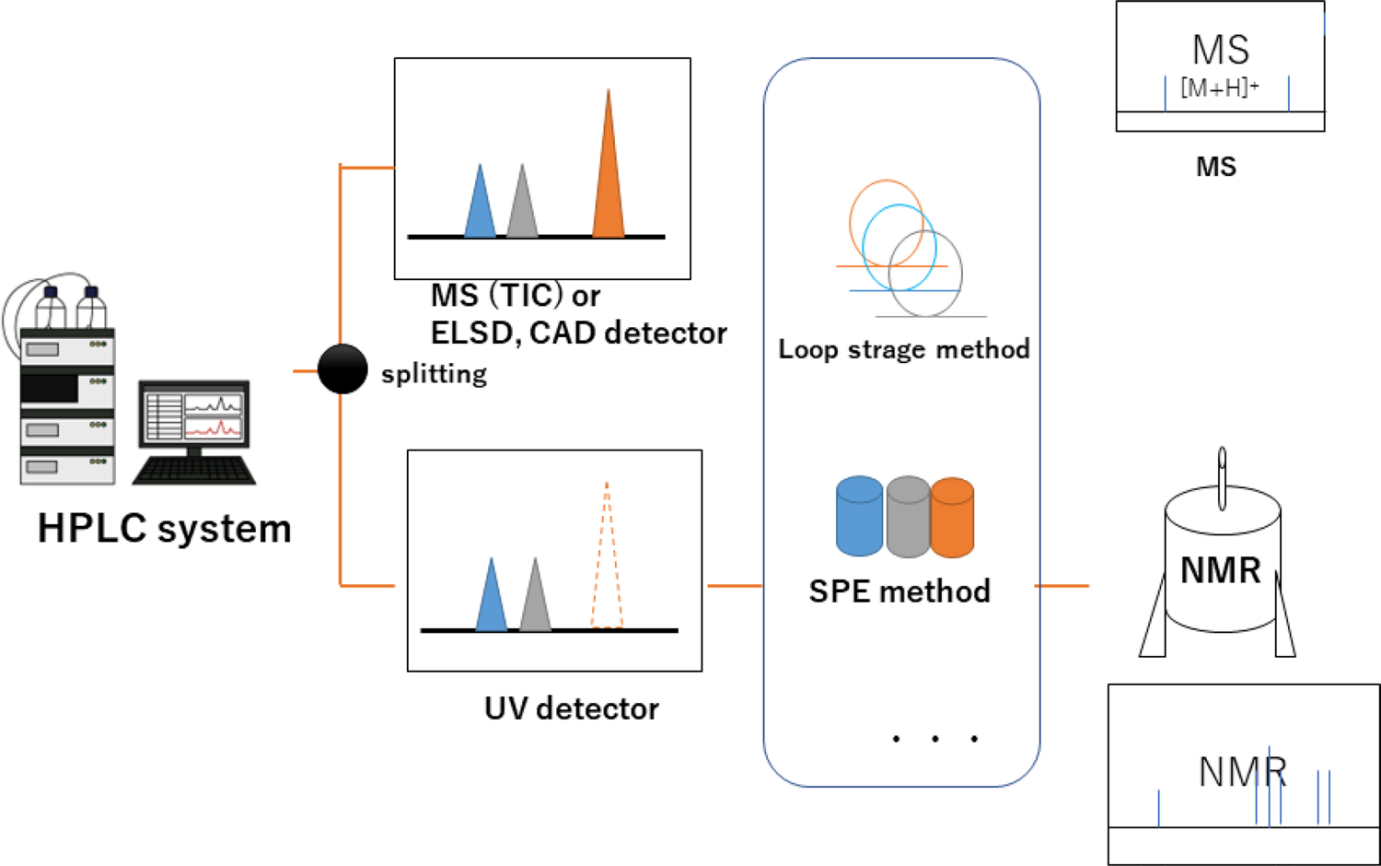
Schematic overview of liquid chromatography-nuclear magnetic resonance (LC-NMR)

**Fig. 2:**
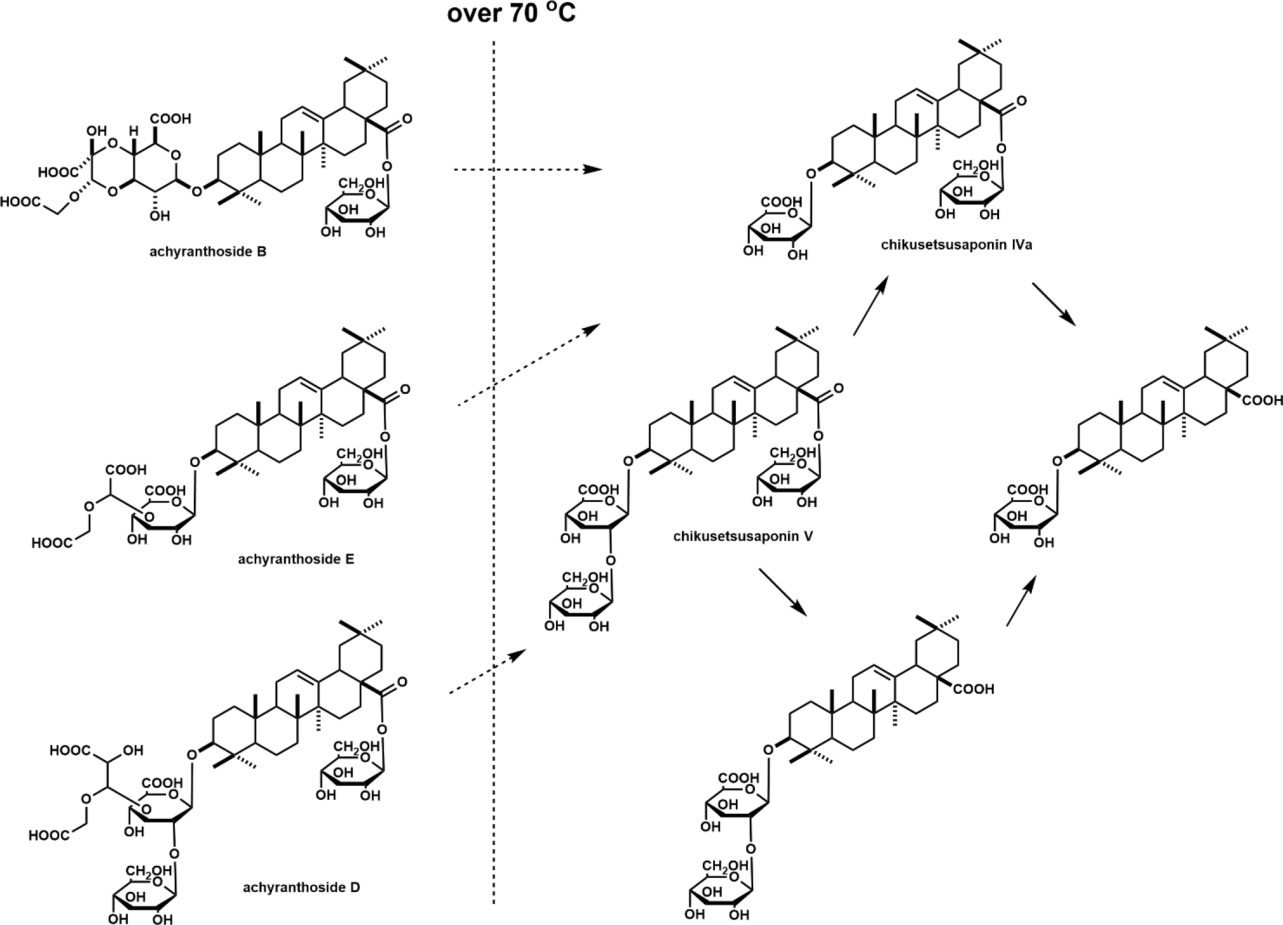
Estimated achyranthoside degradation processes in Achyranthes roots [3]

LC-NMR, in which NMR is directly connected to the HPLC, allows the NMR spectrum of a compound separated by HPLC to be measured directly, enabling the structural analysis of even unstable compounds immediately after separation (Fig. 1). The NMR spectrum is measured after the peaks of the target compounds separated in the HPLC unit are incorporated into the loop; however, for compounds without UV absorption, such as sugars and some terpenes, detecting the peaks of the target compounds is an issue. LC-NMR/MS, which combines LC-NMR with MS, uses a mass spectrometer to detect peaks eluted from HPLC, enabling the detection of compounds with no UV absorption, and the addition of molecular weight information facilitates compound estimation. The addition of NMR also made it possible to determine the structures of isomers that could not be determined by LC–MS. Thus, LC-NMR/MS complements the advantages of mass spectrometry and NMR spectroscopy to enable the structural analysis of samples that are difficult to analyze using conventional LC–MS or LC-NMR alone. Structural changes in the saponin components of *Achyranthes fauriei* roots after harvesting and drying under various temperature conditions were investigated using HPLC and LC-NMR/MS. As a result, it was deduced that significant changes in achyranthosides occur around 70 °C, and that the final degradation process takes place around the drying temperature of 70 °C, as shown in the Fig. 2. LC-NMR has the disadvantage of using either deuterium or a solvent as the mobile phase for the LC when it was first developed, which is costly; however, this disadvantage has been overcome in recent years by coupling it with SPE.

### Determination of unstable components of Leonurus herb using thin-layer chromatography/mass spectrometry (TLC/MS) [7]

**Fig. 3:**
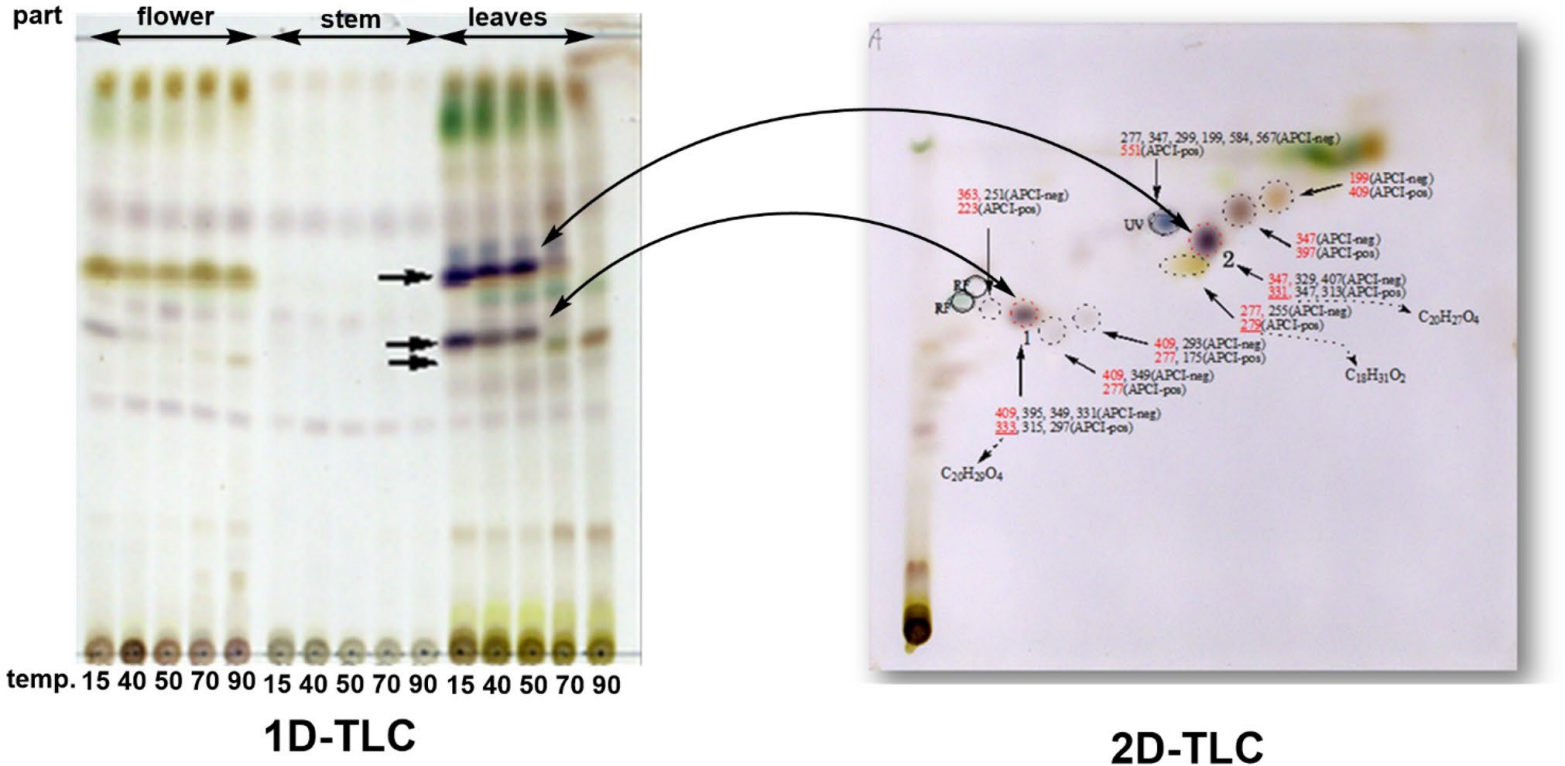
Left: thin-layer chromatography (TLC) of flowers, stems, and leaves at various drying temperatures (arrows indicate spots observed under low-temperature drying conditions for leaves). Right: 2D-TLC/MS results are shown (purple spots were presumed to be diterpenes based on high-resolution MS) [7]. (Modification of Figs. 1 and 3 from Reference 7)

**Fig. 4:**
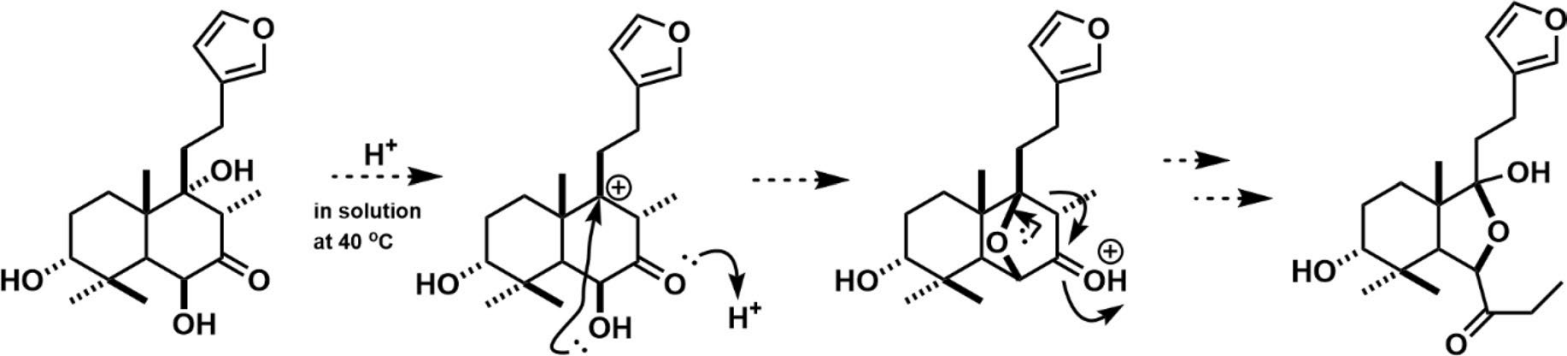
Estimated decomposition processes of the components of *Leonurus japonica* leaves. (Modification of Fig. 7 from Reference 7)

The crude drug, Leonurus herb (益母草), is the above-ground part of *Leonurus japonicus* (Labiatae) during the flowering season, and TLC confirmed that significant differences in the leaf constituents occurred depending on the drying temperature. First, it was assumed that essential oil components, such as monoterpenes, were lost during drying, as this is a constituent of the leaves of Labiatae plants. A clear difference was observed in the GC–MS and TLC comparisons of the extracts immediately after extraction and after approximately six months (refrigerated sealed storage). The lack of a clear correlation between the GC–MS and TLC spots for the target compounds in this case led to a trial of 2D-TLC-MS. The target compound was estimated to be a diterpene based on the high-resolution MS results of the target spot in the 2D-TLC (Fig. 3). The assumption of loss due to drying temperatures caused by volatile components was disproven as diterpenes do not exhibit volatility. The compound was successfully isolated by conducting the entire preparative process at a low temperature, and its structure was eventually revealed by NMR to be a labdane-type diterpene, which decomposed almost completely to a ring-fused compound in approximately three days when heated at 40 °C (Fig. 4). Diterpene compounds in the starting materials are rarely found in marketable crude drugs.

### Change in constituents of Scutellaria root as a consequence of growth years and drying temperature conditions [8]

**Fig. 5:**
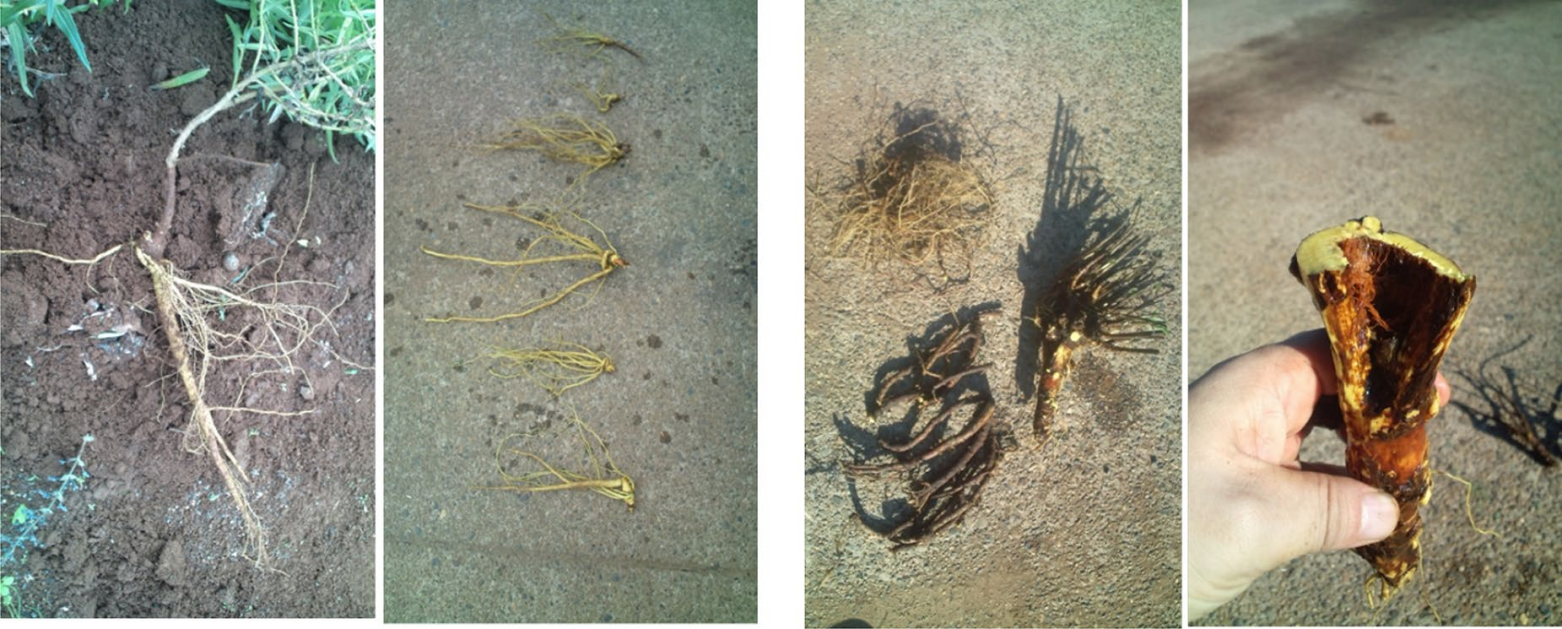
One-year-old root (left) and perennial (right) roots of *Scutellaria baicalensis* [8]

Scutellaria root (黄芩) is the root of *Scutellaria baicalensis* (Labiatae) and has medicinal properties such as anti-inflammatory, promotion of bile secretion, and antipyretic effects (Fig. 5). The main constituent is the flavonoid glycoside baicalin, whereas the other major constituents include baicalein, wogonoside, and wogonin. In preparation and processing, the best type of root has a round shaft with a heavy outer bark that is brownish with a yellow-green interior and is not decayed and hollow. All products marketed in Japan are produced in China. In recent years, the shortage of this plant in China has become a problem, and there has been a switch from wild to cultivated products, often using a method known as "free-range cultivation" to produce properties similar to those of wild plants. It has also been reported that the flavonoid content is higher in cultivated varieties. There is also the issue of the suspected causative crude drug of interstitial pneumonia in the recently problematic Kampo medicine shosaikoto (小柴胡湯); thus, the correlation between the constituents of Scutellaria root and wild and cultivated products is important.

With the processing and preparation of Scutellaria root, the major flavonoid component baicalin is easily hydrolyzed to the aglycones baicalein and glucuronic acid by the enzyme baicalinase, which is present in plants. Therefore, the quality of Scutellaria roots is expected to change significantly during the processing and preparation stages. As mentioned previously, as the cultivation period of Scutellaria roots increases, the roots become old and hollow, resulting in a decline in quality. Therefore, a comparison was made between the composition of 1st-, 2nd-, and 3rd-year roots of the seedling cultivars and the roots from the divided plants.

The contents of the glycosides baicalin and wogonoside tended to decrease with increasing drying temperature when 1st, 2nd, and 3rd year roots were compared. The contents of their respective aglycone parts, baicalein and wogonin, tended to increase with increasing drying temperature, although there was considerable variation in baicalein. No significant differences were observed in the content of the C-glycoside, chrysin-6-*C*-arabinosyl-8-*C*-glucoside, which is presumed to be relatively less susceptible to cleavage by temperature or enzymes at any drying temperature.

### Degradation process of perillaldehyde in Perilla herb and discovery of α-asarone (AS) [9]

**Fig. 6:**
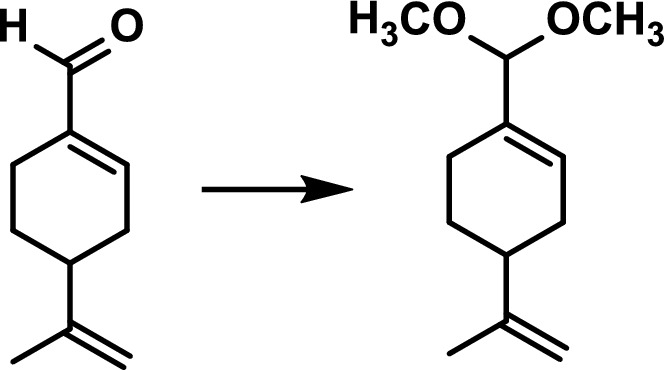
Degradation processes of perillaldehyde

**Fig. 7:**
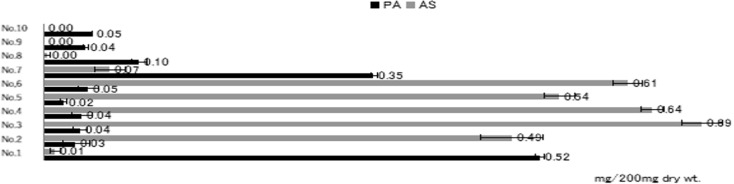
Perillaldehyde (PA) and α-asarone (AS) contents (mg) of Perilla herbs distributed in the Japanese market. (No.1–7; imported (China), No.8–10; domestic). Each value is the mean ± SE (*n* = 3)[9]

Perilla herb (蘇葉) is the leaf and branch tip of *Perilla frutescens* var. *crispa*, and its odor component is perillaldehyde (PA); however, its structure contains an intramolecular aldehyde, which is assumed to rapidly transform into acetal in the presence of alcohol (Fig. 6). The extraction solution and storage conditions of Perilla herb were studied, and finally the identification test method and quantification method in The Japanese Pharmacopoeia were established; however, it was found that some Chinese samples in the process contained little PA and instead contained AS (Fig. 7). Perilla herbs have a phenylpropanoid-rich type (PP-type), but AS was not observed in comparison with domestic PP-type samples. The AS is the* E*-form, while the *Z*-form is a compound that is prohibited as a food additive by the FDA due to its mutagenic properties. This study led to the first detection of this compound in Perilla herbs.

## Study on the uneven distribution of components in plants

### Investigation of the ubiquity of ephedrine alkaloids in Ephedra herb in the plant body [10]

**Fig. 8:**
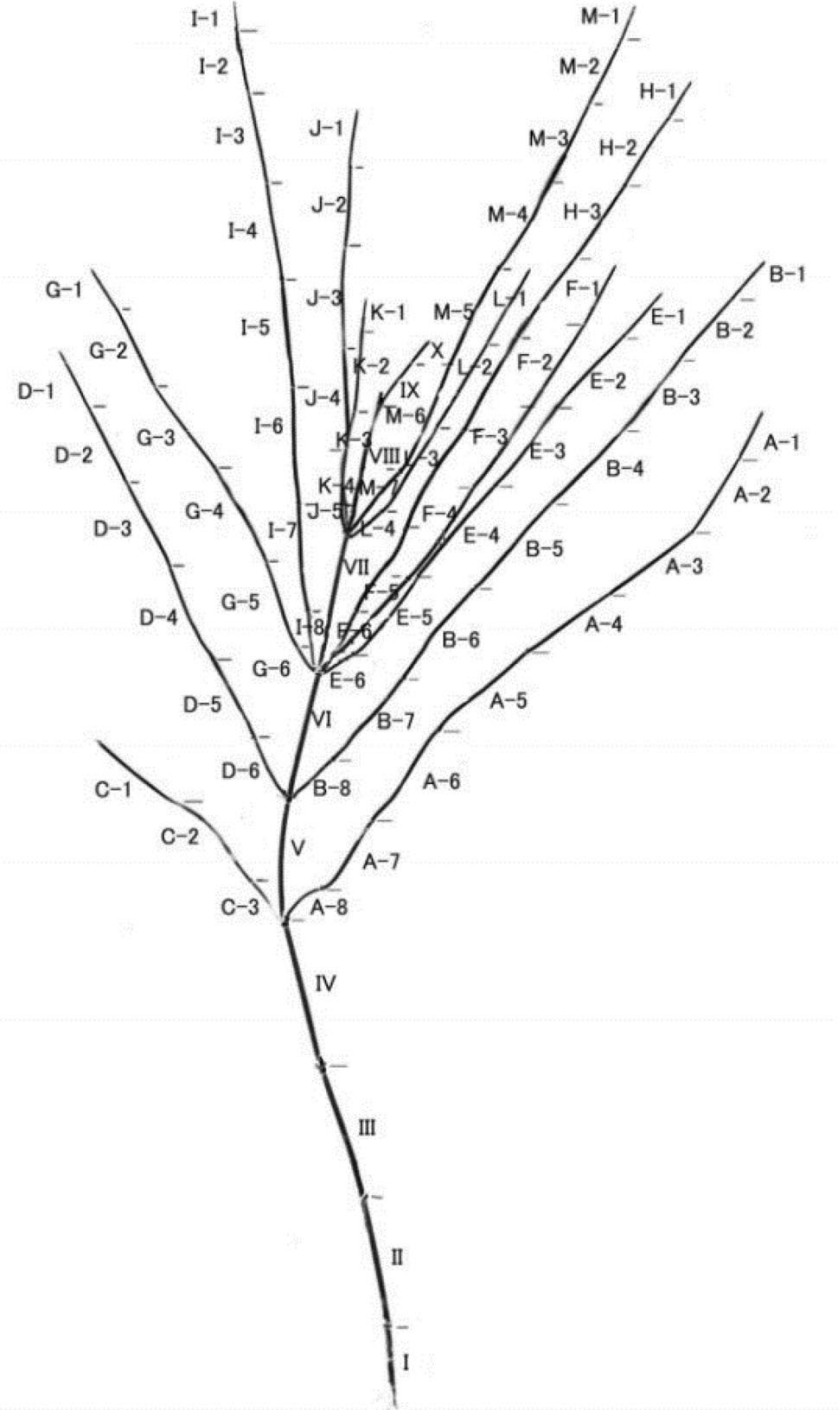
Overall shape of the *Ephedra sinica* whole plant [10]

The crude drug Ephedra herb (麻黄) uses the above-ground part of *Ephedra* sp. In the above-ground parts, the leaves degenerate only to the stem, which is joined by several nodes. Since ancient times, the nodes have been considered to have an antiperspirant effect; thus, Ephedra herb with the nodes removed (去節麻黄) has been considered to be of good quality. Ephedra has current and older branches during its growth, with older branches falling off and being replaced by newer branches. In addition, the distribution of ephedrine in the plant as a whole remains unknown for Ephedra herbs and no guidelines exist for sampling. Ephedrine and pseudoephedrine contents were determined for all internodes of the Ephedra herb using a finger masher and highly sensitive LC–MS analysis. The results showed that in the less-branched *Ephedra sinica,* the apex had lower content, and the internodes closer to the center had higher content. These experimental results could provide useful insights into the sampling of Ephedra herbs, because the contents were lower when only the tip was collected when sampling *Ephedra* sp. (Fig. 8).

## Quality differences between wild and cultivated products

### Comparison between marketed and domestically cultivated products of Saposhnikovia root and rhizome [11]

**Fig. 9:**
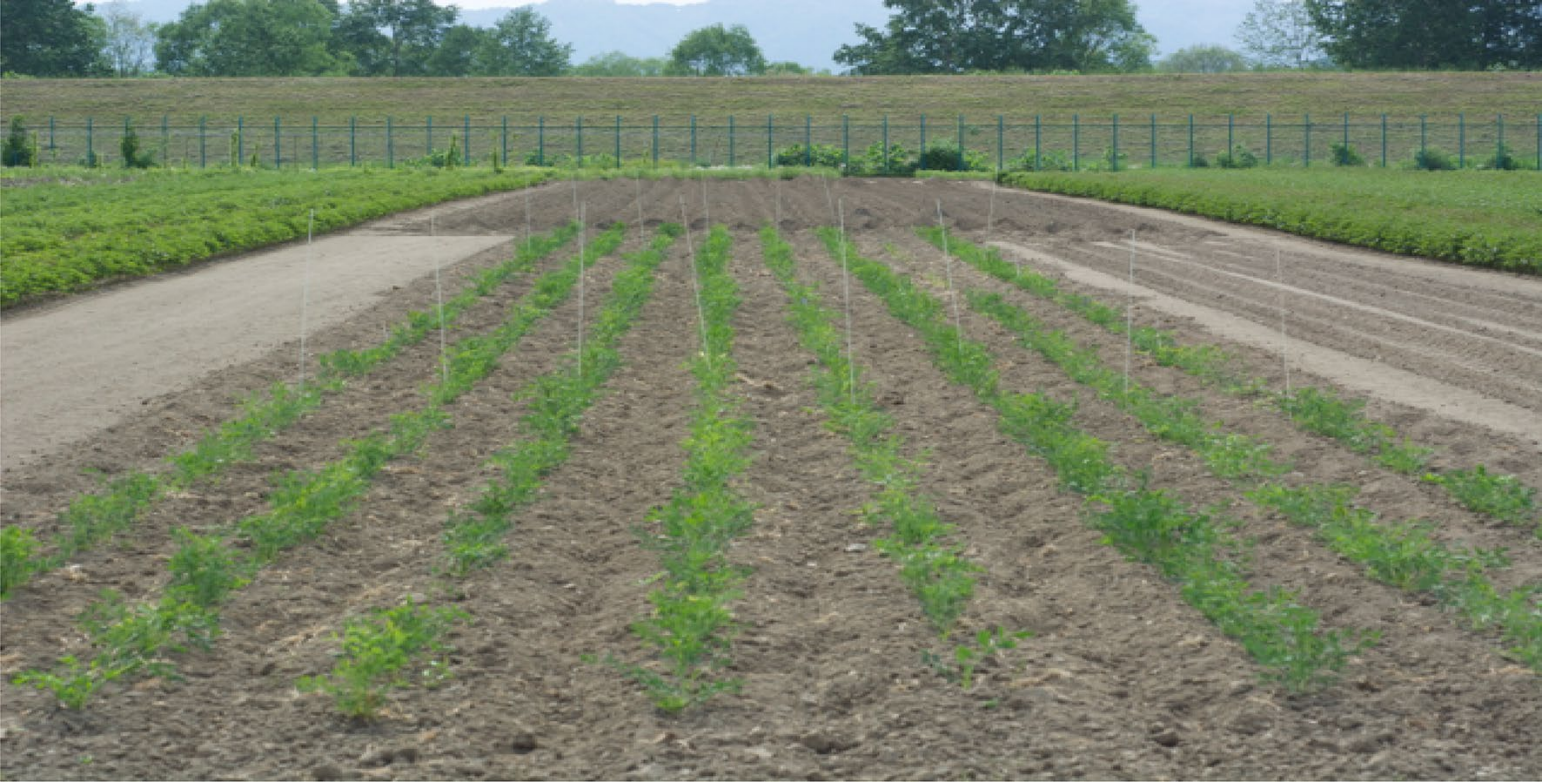
Landscape of *Saposhnikovia divaricata* cultivation fields in Japan

**Fig. 10:**
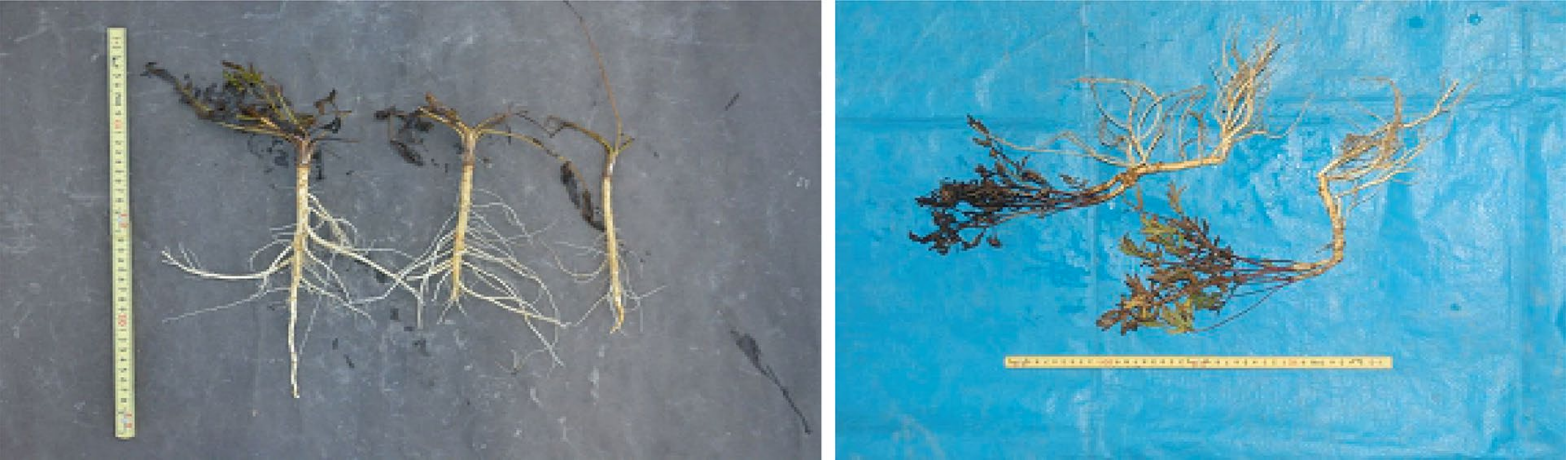
Harvests of domestically cultivated *Saposhnikovia divaricata* (left: original from Beijing, right: original from Heilongjiang Province) [11]

The crude drug, Saposhnikoviae root and rhizome (防風), originates from *Saposhnikovia divaricata* (*Umbelliferae*) (Fig. 9) and has been in increasing demand in recent years as the main ingredient in the diet-promoting Bofutsushosan (防風通聖散). Saposhnikoviae roots and rhizomes are perennial plants, and wild and cultivated products are present in marketed products; 13 marketed products were compared for the content of major components by LC–MS. The content of 4'-*O*-glucosyl-5-*O*-methylvisamminol (GMV), prim-*O*-glucosylcimifugin, cimifugin, and acetylhamaudol was high in the marketed products, but no significant differences were observed between the wild and cultivated products among them. Although 1st-year cultivars are usually considered suitable for harvesting (Fig. 10), the content of the main components in the 1st- and 2nd-year cultivars in domestic cultivation was not sufficient compared to that in the marketed products. It is estimated that a longer growth season is needed before the high content of the components stabilizes in comparison to the six-year-old cultivars in the marketed products.

**Fig. 11:**
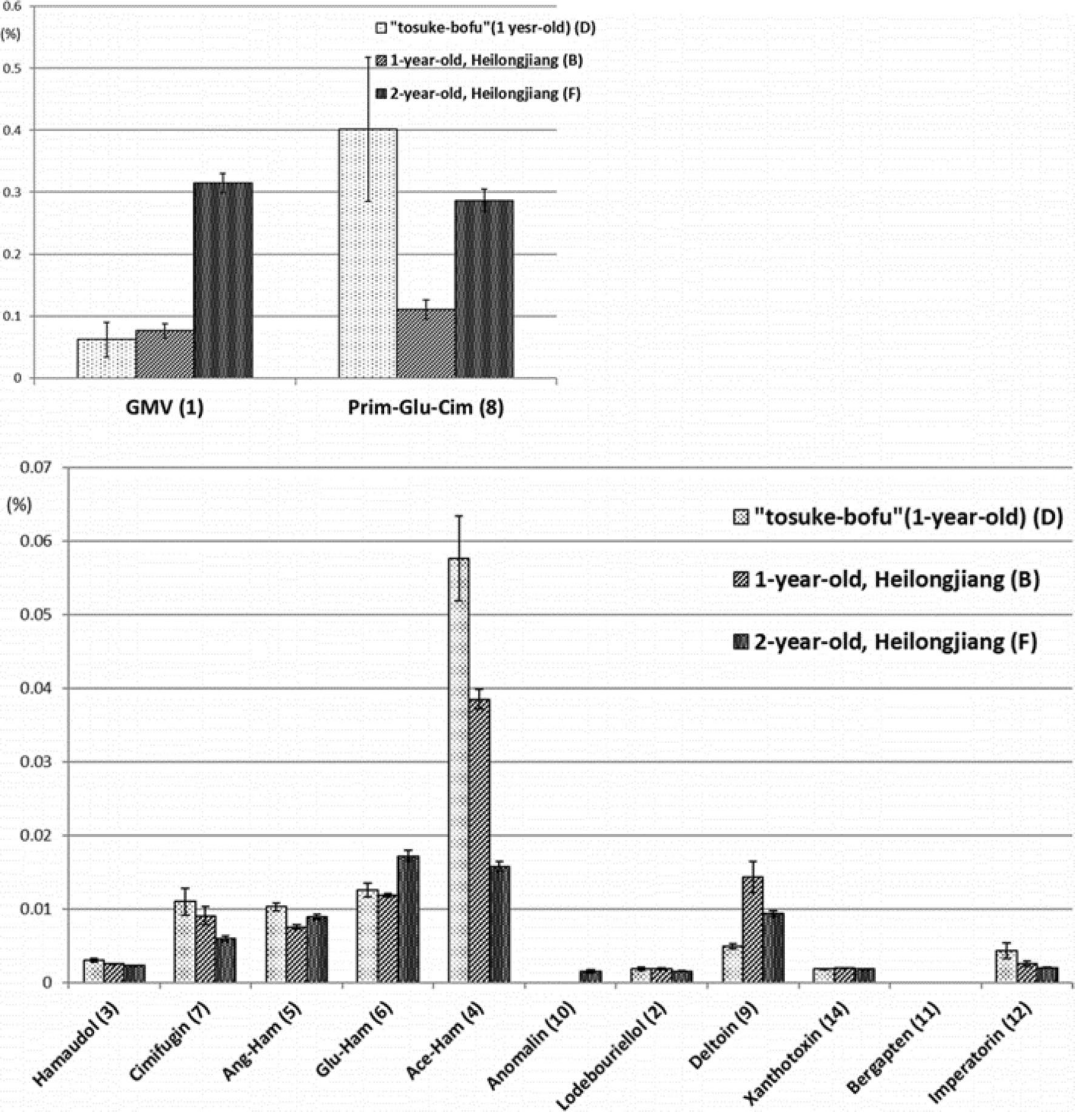
Comparison of constituent contents of Japanese and Chinese cultivars with different growth periods [11]

Comparing the 1st and 2nd year cultivars, the content of glycosides tended to be higher in 2nd year cultivars, while the content of aglycon parts tended to be higher in 1st-year cultivars (Fig. 11). Chromones and furanocoumarins were more abundant in the 1st year. These results suggest that glycosides, such as GMV, are not biosynthesized in sufficient amounts in the 1st year.

**Fig. 12:**
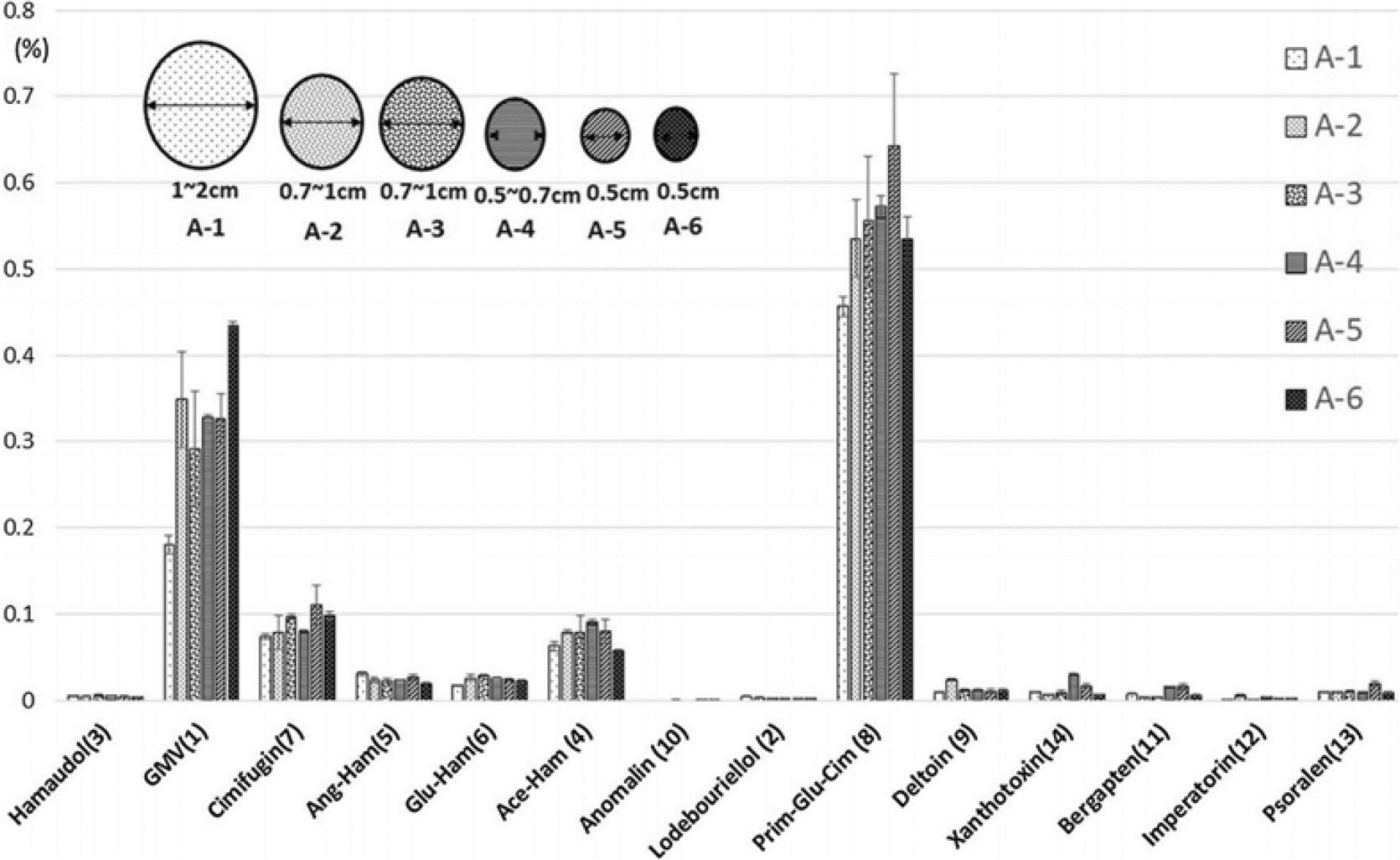
Comparison of constituent according to differences in root diameter[11]

With regard to the differences in root diameter, glycosides accumulated in thin roots, which, as with other crude drugs, were presumed to be more abundant on the root surface (Fig. 12). Comparison of drying temperature conditions showed clear differences in glycoside content between the 50 °C-dried and freeze-dried samples. Although there were some results that could be attributed to enzymatic reactions, as was the case with Scutellaria root, the steric hindrance of the substrate affected the enzymatic degradation, with prim-*O*-glucosylcimifugin, a sugar attached to a primary alcohol, being more likely to undergo enzymatic degradation, while 4'-*O*-glucosyl-5-*O*-methylvisamminol (GMV) bound to tertiary alcohols appeared to be less susceptible. These results may also be due to a balance between enzymatic degradation and thermal desorption.

In the method of processing and preparing the Saposhnikoviae root and rhizome described by Isshiki [12]**,** the root and rhizome are first soaked in water for 15 min after harvesting, drained in a colander, sliced into slices about 0.6 mm thick, and then dried in the sun. How the compositional changes occur in this traditional preparation process remains unresolved.

### Application of multivariate analysis to the quality evaluation of crude drugs

In recent years, multivariate analysis has been used extensively in the field of crude drugs for quality evaluation and identification of bioactive compounds. In addition to LC–MS, spectral data from NMR, NIR [13, 14], and other sources were used as spectral parameters for the multivariate analysis. Examples of the identification of active compounds for the inhibitory effect of nitric oxide (NO) production in ginger and Scutellaria root by multivariate analysis and the analysis of the relationship between the growth environment and components of Ephedra herb and Saposhnikoviae root and rhizome by multivariate analysis are presented.

### Identification of compounds with inhibitory activity on NO production in Ginger and Scutellaria root [15, 16]

**Fig. 13:**
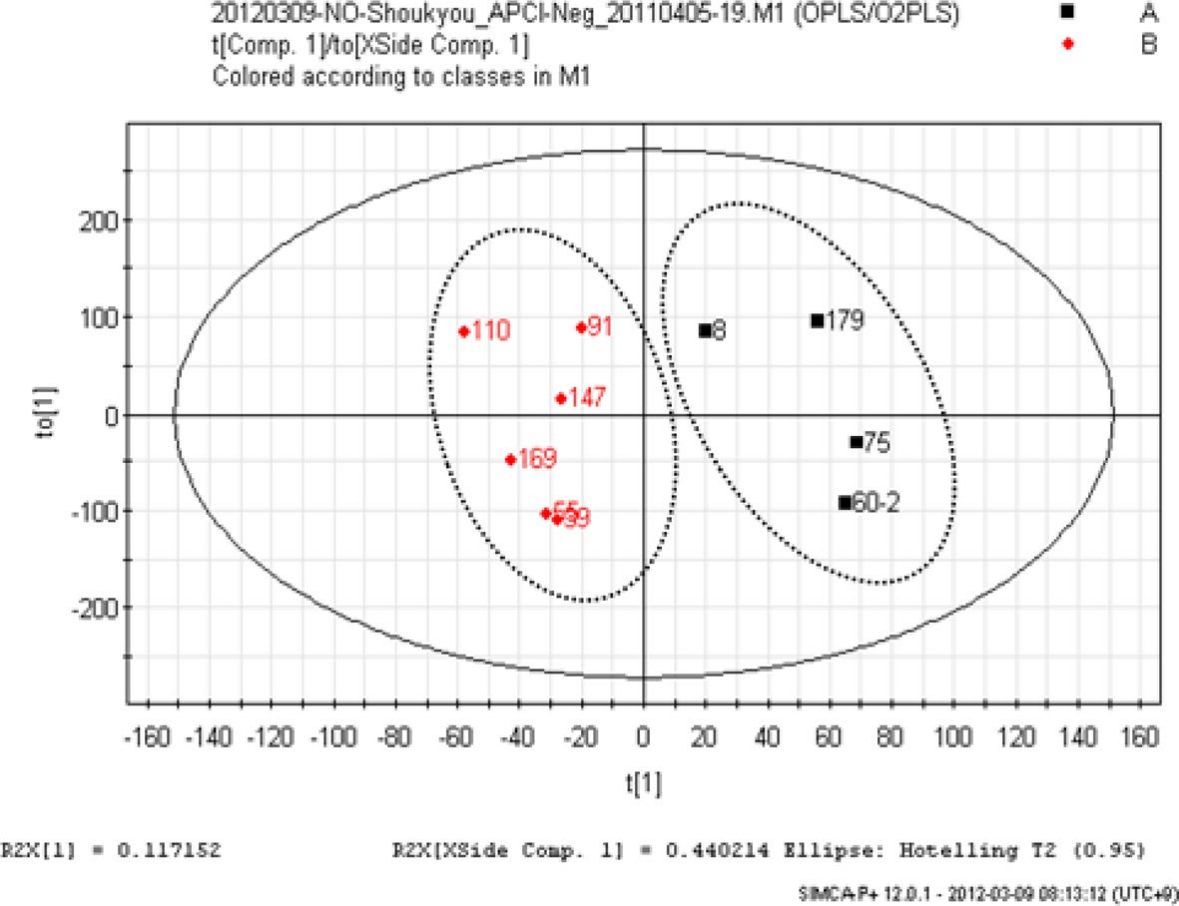
Score plots in orthogonal partial least squares analysis of nitric oxide production inhibitory activity of marketed ginger products[15]

**Fig. 14:**
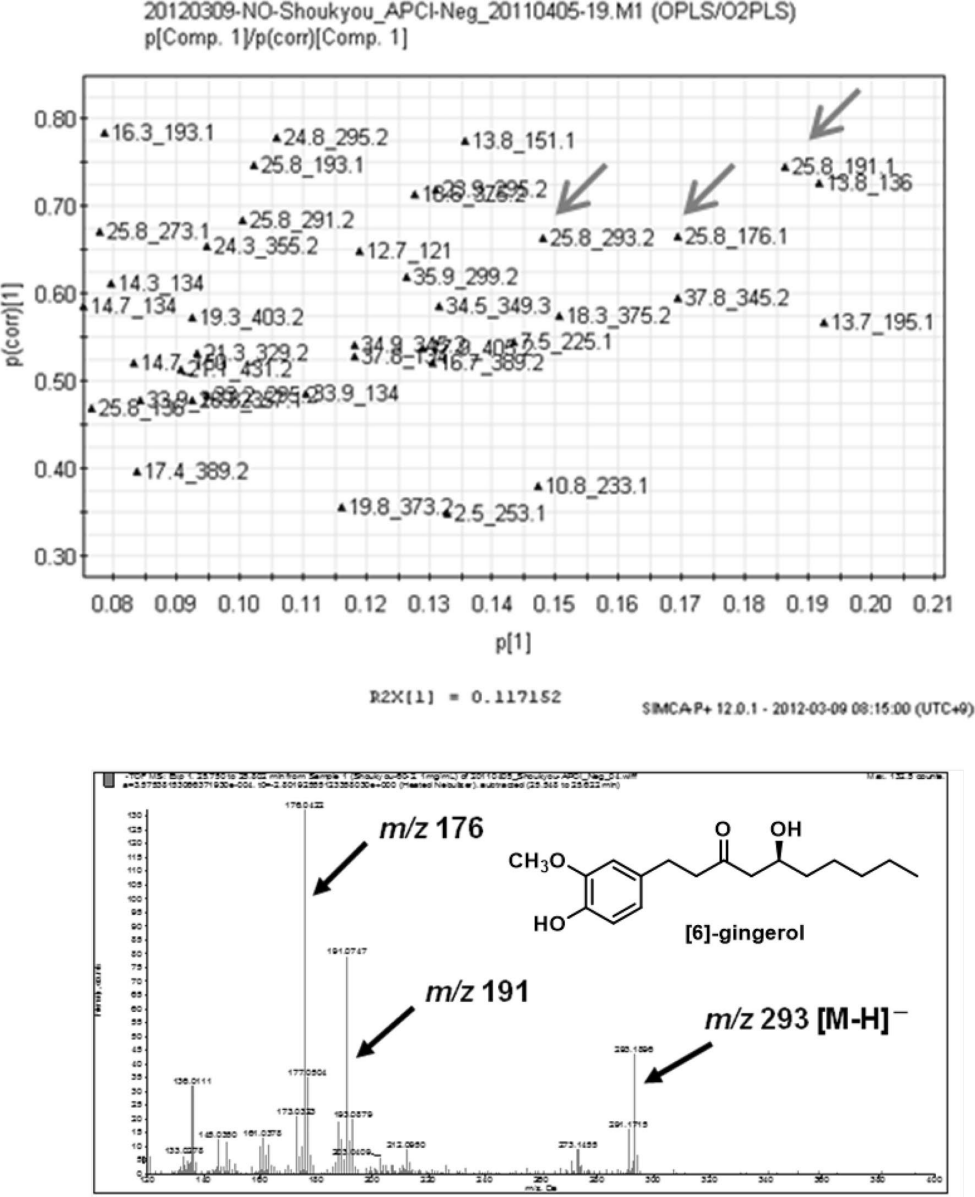
*S*-plot of orthogonal partial least squares analysis and corresponding MS spectra [15]

Active compounds can be identified by extracting marketed crude drugs under identical conditions and analyzing their LC–MS data and biological activity using multivariate analysis. For example, orthogonal partial least squares discriminant analysis (OPLS-DA) of LC–MS data and NO production inhibition activity results were conducted using 10 marketed products of ginger (all from Yunnan), and [6]-gingerol was identified as the contributing component of the strongly active group (Figs. 13 and 14). Subsequent evaluation of the activity of [6]-gingerol alone showed that it inhibited NO production in a concentration-dependent manner. Similarly, multivariate analysis of the LC–MS data and NO production inhibitory activity of 10 marketed crude drugs of Scutellaria root confirmed that wogonoside was the active component. In this case, both OPLS and OPLS-DA confirmed the contributing components.

### Influence of the growing environment on the constituents of Ephedra herb [17]

**Fig.15:**
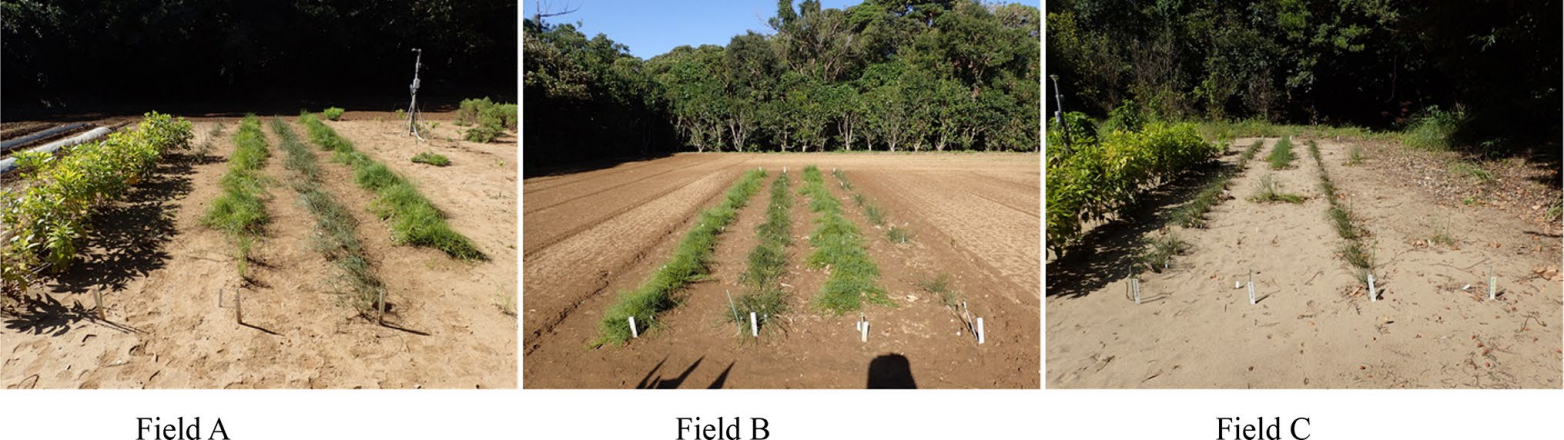
*Ephedra* spp. cultivation fields with three different properties [17]

**Fig. 16:**
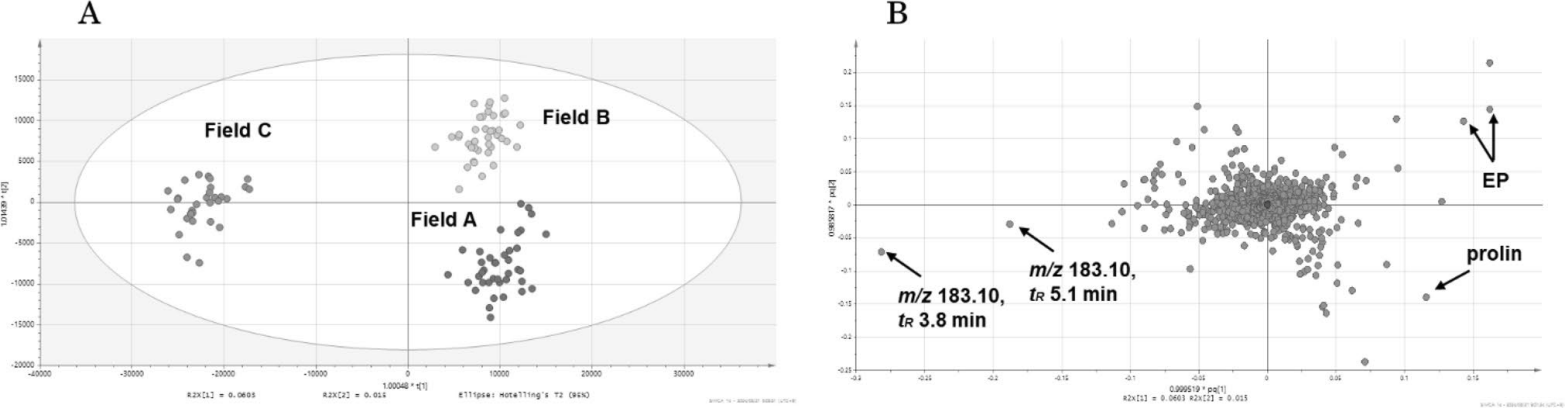
Orthogonal partial least squares discriminant analysis score plot **A** and loading plot **B** for all samples cultivated in fields **A**–**C** [17]

**Fig. 17:**
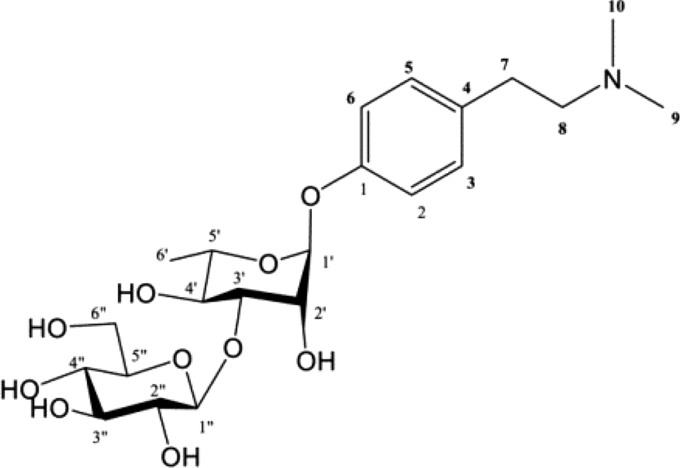
**A** new compound isolated from *Ephedra sinica* [17]

As Ephedra herbs grow in near-desert environments, their relationship with water is likely to be important. *Ephedra* sp. clones were planted and grown in three fields with completely different properties, and their effects on ephedrine and pseudoephedrine contents were investigated (Fig. 15). Nutrient deficiencies and a lack of sunlight in the field led to a decrease in alkaloid content. Multivariate analysis of the affected components between fields showed a clear difference in proline content (Fig. 16). Multivariate analysis of the OPLS-DA comparison between *E. sinica* and EP-13 revealed a characteristic component in *E. sinica*, the novel compound *N,N-*dimethyl-*p*-hydroxyphenylethylamine-*O*-[β-D-glucopyranosyl-(1 → 3)-α-L-rhamnopyranoside] (Fig. 17).

### Comparison of the composition of wild and cultivated products of Saposhnikoviae root and rhizome [11]

**Fig. 18:**
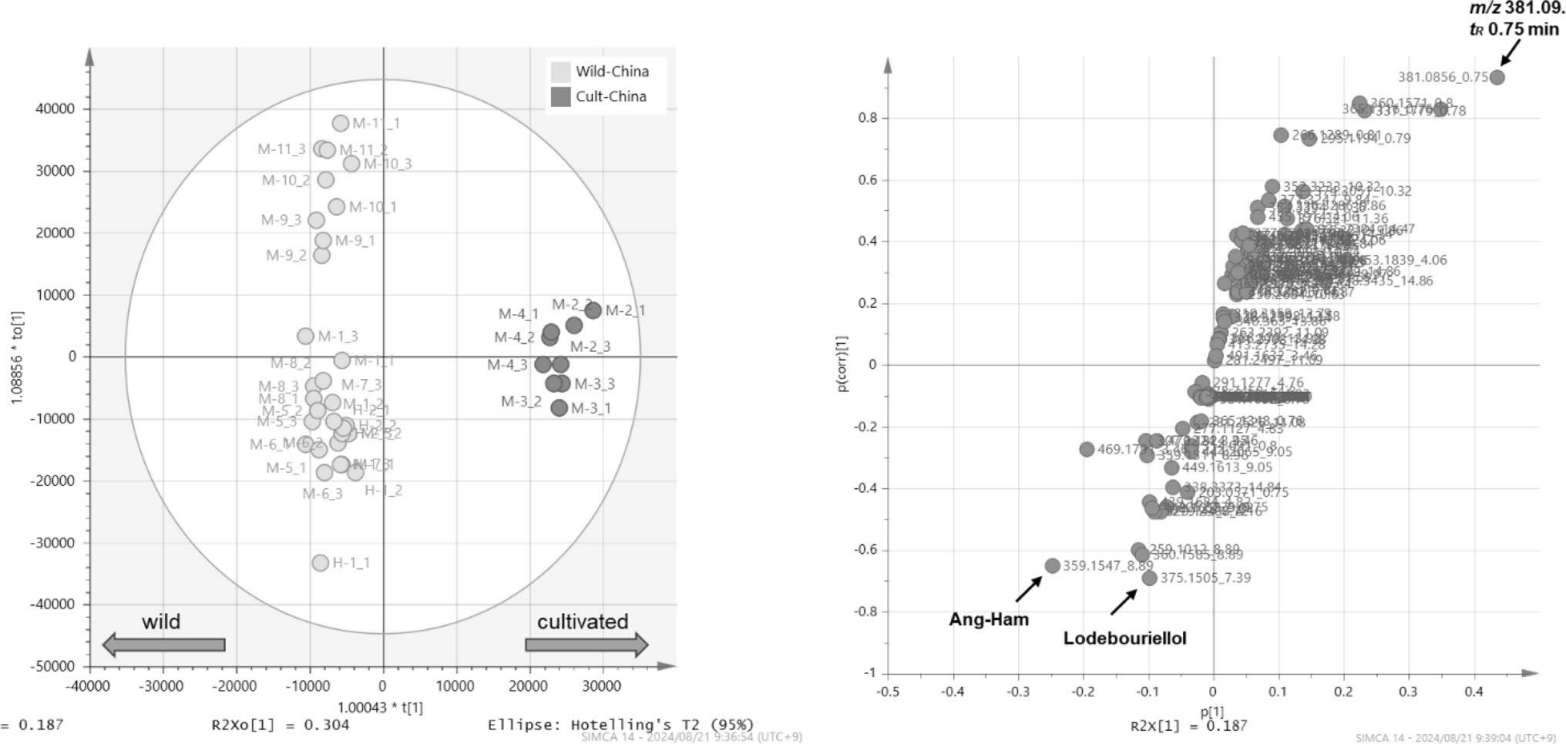
Orthogonal partial least squares discriminant analysis between wild and cultivated Saposhnikoviae Root and Rhizome Japanese market products (left: score plot, right: loading s-plot) [11]

Based on the LC–MS data, the principal component analysis of marketed and domestically grown Saposhnikoviae samples showed that one-year cultivars clearly formed a group distinct from the other samples. The OPLS-DA analysis showed a clear separation between wild and cultivated plants, and the analysis of the contributing components using *S*-plot showed that the content of 3'-*O*-angeloylhamaudol and ledebouriellol was higher in wild plants compared to cultivated plants (Fig. 18). The compound with higher content in the cultivated product was estimated to be 8-hydroxy-5-*O*-β-D-glucopyranosylpsoralen from its MS analysis. Thus, multivariate analysis allowed the compositional differences between the wild and cultivated crude drugs to be clarified.

## Conclusion

The composition of crude drugs can vary significantly not only in their growth environment but also in the processing and preparation stages; therefore, it is necessary to fully examine the factors that cause these compositional variations to assess the quality of these drugs. Drying is an essential process in processing and preparation, but some crude drugs change significantly depending on the temperature at which they are dried; therefore, it is important to control this process. As the compositional content of crude drugs also influences the medicinal properties of the Kampo formulas using them, further clarification of the various factors that cause compositional changes is needed. In addition, as crude drug components are multicomponent systems, focusing on only one component does not provide a holistic view. Therefore, multivariate analysis is effective for evaluating the quality of crude drugs. It was possible to evaluate the differences in composition between crude drugs from various aspects, such as between the original plants, number of years of growth, and wild and cultivated products. In the future, multivariate analyses of crude drug components are expected to be developed in a wider range of fields, such as the analysis of new spectral data and Kampo formulas.
